# The Relationship Between Hematological Parameters and Mortality in Cardiovascular Patients With Postcardiac Arrest Syndrome

**DOI:** 10.7759/cureus.6478

**Published:** 2019-12-27

**Authors:** Mehmet K Erol, Nazım Kankılıc, Firdevs Kaya, Ahmet Atlas, Veli Fahri Pehlivan, Basak Pehlivan

**Affiliations:** 1 Anesthesiology, Harran University, Sanliurfa, TUR; 2 Cardiovascular Surgery, Harran University, Sanliurfa, TUR

**Keywords:** cardiac arrest, post-cardiac arrest syndrome, red blood cell distribution width, mean cell hemoglobin concentration

## Abstract

Background: Post-cardiac arrest syndrome is the insufficiency of cardiac and cerebral functions caused by ischemia after sudden cardiac arrest. We aimed to determine the hematological parameters associated with mortality in the intensive care follow-up of patients with post-cardiac arrest syndrome.

Methods: The hematological parameters of 285 cardiovascular patients who were admitted to the emergency department of Harran University Medical Faculty between 2013 and 2018 and followed up in the intensive care unit with post-cardiac arrest syndrome were examined. A total of 85 patients were included in the study. These parameters were recorded as the time of arrival to the emergency department (0 hour) and hematological parameters at the 24^th^ and 48^th^ hours of intensive care follow-up.

Results: In the mortality group, albumin (P:0.030), hemoglobin (Hg) (P: 0.049), and hematocrit (HCT) (P: 0.020) values ​​in the blood parameters, at the time of admission to the emergency department, were significantly lower than those in the survival group. Red blood cell distribution width (RDW) (P: 0.009) and urea (P <0.001) values ​​at the time of arrival were higher than the survival group. In the 24^th^ and 48^th^ hours, mean hemoglobin (MCHC) (P <0.05) values ​​were lower and RDW (P <0.05) values ​​were higher in the mortality group compared to the survival group.

Conclusions: In this retrospective validation, low albumin, Hg, HCT, MCHC, and high RDW and urea levels may increase mortality in cardiovascular patients who develop post-cardiac arrest syndrome within the first 48 hours. Correcting these values ​​early may reduce mortality.

## Introduction

Cardiac arrest is a condition characterized by the sudden loss of consciousness caused by inadequate cerebral blood flow and sudden stop of blood circulation as a result of the inability of the heart to contract effectively during systole [1-2]. The number of sudden cardiac deaths per year is defined as 1 in about 1000 people [3]. Cardiac arrest is often associated with mortality and morbidity. Mostly seen in individuals with underlying cardiovascular disease [4-5]. The cause of morbidity and mortality after cardiac arrest is the inadequacy of cerebral and cardiac functions due to prolonged whole-body ischemia called post-cardiac arrest syndrome (PCAS) [6]. Acute tubular necrosis, traumatic liver injury and shock liver, ischemic colitis, pneumothorax, bone marrow embolism, cholesterol embolism, rib fractures, ventilator-associated pneumonia, sepsis, and many other systemic serious complications may develop after cardiopulmonary resuscitation. Post-resuscitation syndrome caused by whole-body ischemia-reperfusion injury is a sepsis-like condition and constitutes an inflammatory response characterized by myocardial and adrenal dysfunction and hyper coagulopathy. This leads to hypoperfusion and multiorgan dysfunction [1-4]. Intensive care units are the most important step in the follow-up and survival of critically ill patients with this type of PCAS. It is important to determine the parameters predicting mortality in ICU patients. It is important to make periodic improvements by adjusting each variable to be included in these parameters [7]. The aim of this study was to retrospectively evaluate the relationship between mortality and blood parameters in the intensive care unit of cardiovascular patients who were admitted to the emergency department with sudden cardiac arrest.

## Materials and methods

Two hundred and eighty-five cardiovascular patients admitted to Harran University Medical Faculty emergency department between 2013-2018 were evaluated. The data of the patients were analyzed retrospectively from hospital records and hospital data processing system. Patients with malignancy, under 18 years of age, infection, recent major surgery, chronic renal failure, chronic obstructive pulmonary disease, hematologic diseases, post-cardiopulmonary resuscitation in the emergency department who are ex, cardiopulmonary resuscitation (CPR) duration greater than 40 minutes were excluded from the study. After exclusion, 85 patients were included. Demographic data and length of hospitalization were recorded. Complete blood counts, biochemical analyzes and blood gas values ​​were recorded in the intensive care units when the patients arrived at the emergency department (0 h) (T0), at the 24th hour (T1), and 48th hour (T2). All blood values ​​were statistically compared between the mortality and survival groups (Mortality group: Group 1, Survival - ICU Discharge group: Group 2). All steps of the study conformed to the guidelines of the Declaration of Helsinki, and informed consent was obtained from all the participants.

Statistical Package for the Social Sciences; version 18.0 (SPSS Inc., Chicago, IL) program was used for the statistical analysis. The Kolmogorov-Smirnov test was used to evaluate the normal distribution of continuous variables. The Mann-Whitney U test and Student’s t-test were used to compare variables. Pearson’s and Spearman’s correlation tests were used for the correlation analysis. The mean values of all the parameters are presented, together with their standard deviations. A p-value of less than 0.05 was considered statistically significant.

## Results

The mean age of the patients included in the study was 60.50 ± 18.90 years. Of the 85 patients, 44 (51.7%) were male and 41 (48.3%) were female. 27 patients had hypertension, 18 patients had diabetes and hypertension, 30 patients had coronary artery disease, 10 patients had valvular heart disease. In the comparison between the groups, it was seen that age was significantly higher in the Mortality group (Group-1: 62,30±18,93, Group-2: 50,53±15,870) (P:0,038). At the time of arrival (0th hour) of the patients, albumin (P: 0.030), hematocrit (HCT) (P: 0.020), hemoglobin (Hg) (P: 0.049), red blood cell distribution width (RDW) (p: 0.009), urea-blood urea nitrogen (BUN) (p <0.001) values ​​were found to be statistically significant between the two groups. Hemoglobin (Hg) (P: 0.041) and mean cell hemoglobin concentration (MCHC) (P: 0.039) showed significant differences in the 24th hour. At 48th hour, RDW (P: 0.009) and MCHC (P: 0.028) values ​​were significant parameters. Blood parameter analyses between groups were summarized in Table 1. The data was normally distributed and significant differences between groups are revealed with correlation analysis. The relationship between hematological parameters and mortality in cardiovascular patients with postcardiac arrest syndrome was investigated. Urea (P: 0.000, r: 0.429) values at arrival time ​​were positively correlated with age, and albumin (p: 0.027, r: -0.245) values at arrival time ​​were negatively correlated with age. Other correlation data are summarized in Tables 2-4. In the analysis, RDW values ​​at arrival time and MCHC values of the 24-48th hour ​​were significantly related with BUN, albumin, HCT and Hg values (P <0,001).

**Table 1:** Blood parameter analyses between groups HCT: hematocrit; MPV: mean platelet volume; MCHC: mean corpuscular hemoglobin concentration; MCV: mean corpuscular volume; RDW: red cell distribution width; CRP: C-reactive protein.

	Hospital Mortality Group (n:72)	ICU Discharge Group (n:13)	P
Age (Year)	62,30±18,93	50,53±15,870	0,038
Albumin (g/dl) (O.h)	2,98±0,742	3,52±0,881	0,030
Albumin (g/dl) (24.h)	2,61±0,63	2,90±0,89	0,320
Albumin (g/dl) (48.h)	2,43±0,49	2,70±0,62	0,269
HCT (%) (O.h)	39,51±9,54	46,028±6,04	0,020
HCT (%) (24.h)	38,57±9,44	43,92±9,10	0,115
HCT (%) (48.h)	35,72±8,34	37,82±7,75	0,487
Hemoglobin (g/dl) (O.h)	12,57±2,77	14,25±2,90	0,049
Hemoglobin (g/dl) (24.h)	12,09±2,93	14,27±3,02	0,041
Hemoglobin (g/dl) (48.h)	11,09±2,53	12,29±2,75	0,201
MPV (fL)(O.h)	7,38±1,36	6,65±2,11	0,249
MPV (fL)(24.h)	7,75±1,45	7,31±1,42	0,406
MPV (fL) (48.h)	7,84±1,39	8,58±1,73	0,256
MCHC (g/dl) (O.h)	31,60±1,88	30,98±4,79	0,419
MCHC (g/dl) (24.h)	31,38±1,46	32,47±1,37	0,039
MCHC (g/dl) (48.h)	31,13±1,58	32,40±1,32	0,028
MCV (fL) (0.h)	88,686±9,45	90,42±5,73	0,524
MCV (fL) (24.h)	88,23±13,52	84,91±10,25	0,127
MCV (fL) (48.h)	87,76±10,17	87,16±6,72	0,866
RDW (%) (0.h)	13,63 ± 2,94	12,24± 1,98	0,009
RDW (%) (24.h)	14,06±2,63	13,23±3,70	0,074
RDW (%) (48.h)	13,71±2,02	11,83±1,49	0,012
Urea(g/dl) (0.h)	61,54±41,84	29,75 ± 21,48	0,000
Urea(g/dl) (24.h)	88,70±52,40	57,79±29,15	0,051
Urea(g/dl) (48.h)	98,15±68,72	65,27±29,44	0,145
Creatinine (g/dl) (0.h)	1,56±1,35	1,16±0,35	0,705
Creatinine (g/dl) (24.h)	2,07±1,63	1,38±1,56	0,183
Creatinine (g/dl) (48.h)	2,10±2,08	2,07±1,63	0,124
Uric acid(mg/dl)(0.h)	8,50±3,29	6,68±2,14	0,071
Uric acid(mg/dl) (24.h)	8,87±3,16	16,45±11,95	0,028
Uric acid(mg/dl)(48.h)	7,16±3,52	3,82±2,39	0,149
CRP (mg/dl) (0.h)	6,34±16,98	2,55±7,08	0,058
CRP (mg/dl) (24.h)	12,05±8,86	7,90±6,09	0,270
CRP (mg/dl) (48.h)	13,44±9,13	15,52±5,60	0,567

**Table 2:** Correlation analyses (T0) HCT: hematocrit; RDW: red cell distribution width; MCHC: mean corpuscular hemoglobin concentration.

	Albumin (g/dl)	HCT (%)	Hemoglobin (g/dl)	RDW (%)	Creatinine (g/dl)	MCHC (g/dl)
Urea(g/dl)	p:0.000 r:-0,478	p:0.001 r:-0,343	p:0.001 r:-0,346	p:0.000, r:0,418	p:0.000 r:0,481	P>0,05
HCT (%)	p:0.000 r:0,597	-	p:0.000 r:0,910	p:0.000 r:-0,415	P>0,05	P>0,05
Hemoglobin (g/dl)	p:0.000 r:0,577	p:0.000 r:0,910	-	p:0.000 r:-0,489	P>0,05	p:0.006 r:0,294
Albumin (g/dl)	-	p:0.000 r:0,597	p:0.000 r:0,577	p:0.001 r:-0,358	P>0,05	P>0,05

**Table 3:** Correlation analyses (T1) HCT: hematocrit; RDW: red cell distribution width; MCHC: mean corpuscular hemoglobin concentration.

	Albumin (g/dl)	HCT (%)	RDW (%)	Creatinine (g/dl)	Urea(g/dl)
Hemoglobin (g/dl)	p:0.000 r:0,616	p:0.000 r:0,979	p:0.002 r:-0,357	P>0,05	p:0.030 r:-0,255
MCHC (g/dl)	P>0,05	P>0,05	p:0.000 r:-0,403	p:0.023 r:-0,265	p:0.040 r:-0,242

**Table 4:** Correlation analyses (T2) HCT: hematocrit; RDW: red cell distribution width; MCHC: mean corpuscular hemoglobin concentration.

	Albumin (g/dl)	HCT (%)	Hemoglbin (g/dl)	Urea(g/dl)	Creatinine (g/dl)	MCHC (g/dl)	RDW (%)
RDW (%)	P:0.049 r:-0,292	P :0.023 r:-0,299	P :0.002 r:-0,392	P >0,05	P >0,05	P :0.001 r:-0,437	-
MCHC (g/dl)	P >0,05	P >0,05	P >0,05	P :0.004 r:-0,373	P :0.016 r:-0,318	-	P :0.001 r:-0,437

## Discussion

Albumin binds many substances in the body, transports them and removes free radicals. It can capture multiple reactive oxygen products and reactive nitrogen products. It is also known that it increases anti-inflammatory cytokines and is an important extracellular antioxidant [8]. In addition, it plays an important role in the inhibition of platelet activation and aggregation and vascular endothelial apoptosis [9]. Studies have shown a relationship between hypoalbuminemia and increased mortality and morbidity [10-11]. It is also mentioned to be an independent predictor of early heart failure [10]. In our study, the blood values ​​obtained at the first admission of the patients who had mortality, it was observed that albumin values ​​were low (P: 0.030) (Table 1). We think that this may be due to a global systemic ischemia-reperfusion response and subsequent systemic inflammatory response in patients with PCAS. This leads to a degradation resistant to vasoregulation and increased coagulation. Albumin accounts for about 80% of colloid osmotic pressure, thus increasing the vascular hyper-permeability of the inflammatory state, leading to a decrease in oncotic pressure in patients with hypoalbuminemia and thus a reduction in intravascular volume. This creates insufficient blood flow to vital organs [12]. Therefore, low levels of albumin at the time of arrival of patients with PCAS will increase end-organ damage. Early correction of this value will reduce this damage. In animal studies as evidence of this, albumin supplementation improves cerebral blood flow in cardiac arrest and resuscitation models [13].

Anisocytosis has traditionally been defined as the heterogeneity of erythrocyte volumes. The RDW, which expresses anisocytosis, is a laboratory index that shows the change in red blood cell size in the whole blood count. RDW is calculated by dividing the volume of red blood cells (RBC) by the mean corpuscular volume (MCV) [13]. Studies have shown that RDW is associated with blood inflammation markers such as interleukin-6 and C-reactive protein (CRP). In addition, oxidative stress disrupts erythropoiesis and increases anisocytosis and it has been shown that it causes membrane damage leading to RDW increase and reduces circulating red blood cell half-life [14-15]. High RDW has been associated with poor prognosis in various diseases such as pneumonia, cardiovascular diseases, pulmonary embolism, cancer and cerebral infarction [16]. Recently, RDW has been evaluated as a prognostic marker for short and long term mortality in critically ill patients [17]. Similarly, in our study, high RDW (P: 0.009), low Hg (P: 0.049), low HCT (P:0.020), and a decrease in MCHC (P: 0.039) at 24th hours were observed in patients with mortality (Table 1). It is thought that this is due to the development of systemic inflammation response in patients with sudden cardiac arrest and PCAS and oxidative stress reduces RBC survival and affects bone marrow functions and premature RBCs cause an increase in RDW with increased release of peripheral circulation. Similarly, the decrease in RBC survival can be explained by the same mechanism by causing hemolysis and a related decrease in Hg, HCT and MCHC values [18]. To support this, proinflammatory cytokines have been shown to inhibit erythropoietin-induced erythrocyte maturation and proliferation and reduce erythropoietin receptor expression associated with increased RDW [19].

Glomerular filtration rate (GFR), urea-BUN, and creatinine (Cr) were used to evaluate patients' renal function and fluid status [20]. Both Cr and urea are filtered from the glomerulus due to their small molecular size. Cr is rarely absorbed from the tubules and excreted in the urine from the body. Urea is absorbed from the tubules. Urea-BUN also has an important role in glomerular water balance. Urea-BUN is a marker for both neurohormonal activity and renal function because reabsorption of urea varies not only with the renal route but also with endocrine disorders [21]. High urea levels are known to be associated with mortality in cardiovascular disorders [22]. In our study, urea values ​​at the time of admission were higher in patients with PCAS in the group associated with mortality (61,54±41,84), significantly lower in the group without mortality (29,75± 21,48) (P <0.05). In addition, it was observed that urea values ​​at 24th and 48th hours were higher in the mortality group although not statistically significant (Figure 3). However, the relationship between Cr values ​​and mortality has not been fully explained. Cr values ​​were similar in both groups from the time of arrival (P>0.05) (Table 1). The reason for this is thought to be the activation of renin-angiotensin-aldosterone and sympathetic nervous systems caused by low cardiac output, renal hypoperfusion, decreased GFR and renal hypoperfusion in patients with PCAS [22]. This also affects serum Cr levels. However, serum Cr levels may also vary for a variety of reasons (age, sex, muscle mass, and diet, etc.) [23]. This made us think that urea-BUN levels in PCAS patients may provide more useful information on mortality compared to Cr.

## Conclusions

Urea-BUN, albumin, HCT, Hg, MCHC, RDW values ​​should be carefully monitored for the first 48 hours in patients with PCAS with cardiac disease. Low Hg, HCT, MCHC and high RDW and urea levels may increase mortality in these patients; we think that early correction of these values ​​may reduce mortality. Further studies on patients with PCAS in the future will increase our knowledge on this subject.
